# Radiographic Morphometric Measurements of the Donkey’s Distal Hind Limb

**DOI:** 10.3390/ani15010022

**Published:** 2024-12-25

**Authors:** Kyra Schaus, Juliana Wacker, Anabell Jandowsky, Kathrin Büttner, Michael Röcken, Claus Peter Bartmann

**Affiliations:** 1 Clinic for Equine Surgery and Orthopedics, Justus-Liebig-University Giessen, 35392 Giessen, Germany; 2 Arche Warder—Zentrum für alte Haus- und Nutztierrassen e.V., Langwedeler Weg 11, 24646 Warder, Germany; 3 Unit for Biomathematics and Data Processing, Justus-Liebig-University Giessen, 35392 Giessen, Germany

**Keywords:** donkey, hindlimb, radiograph, X-ray, measurement, hoof, toe, physiology, equid, database

## Abstract

Morphometric measurements of the toe serve as a reference for the objective assessment of the presence of a pathological condition and as a guide for the correct trimming of the hooves. Therefore, the anatomical differences between horses and donkeys oblige an autonomous database with reference values for the donkey. The aim of this study was therefore to provide readings of the distal hind limb on radiographs of donkeys. Radiographs of the distal hind limb in two projections of 41 generally sound donkeys without lameness in walk were taken after previous hoof trimming and preparation according to a standardized radiographic procedure. A total of 16 lengths and 9 angles were measured and statistically analyzed in each hoof. The dorsal hoof wall is on average 70 to 80 mm long and angled at 55°; the coffin bone runs parallel to the hoof wall. The average founder distance is 17 mm, and the mean sole thickness is 14 mm. The heel angle averages 52°, which is slightly flatter than the hoof wall angle. Correlations with weight and height are observed. Concluding, the morphometric measurements confirm the differences between horses’ reported normal anatomy and the presented donkeys. Disorders can be detected by applying the measurements.

## Simple Summary

Donkeys and horses are close relatives of the equid species but nevertheless exhibit relevant differences in body structure, particularly in the feet. The state of health of the hooves and their bony structures can be objectively assessed using distance and angular measurements on radiographs. The measured values determined for the horse are often used analogously for the donkey, although it must be assumed that their transferability is limited. Due to a lack of data on the hind feet of donkeys, radiographs of this region from 41 generally sound donkeys were analyzed to provide a database for improving medical care and hoof treatment. The results show that donkeys have steeper hind hooves compared to horses and that the coffin bone is located more distally in the hoof capsule. Furthermore, positive relationships were found between all measurement variables and weight, with the length measurements being more dependent on weight than the angular measurements. There are hardly any clinically relevant differences between left and right. Further studies with larger numbers of donkeys and more accurate examination methods are required for validation and supplementation.

## 1. Introduction

The hoof condition has been shown to have a significant impact on the welfare and health of donkeys [1,2,3]. In a British donkey population studied over a seven-year period, 44.8% of euthanized cases were diagnosed with foot disorders at necropsy, which are therefore of high clinical relevance in this equid species [4]. Links between neglected hoof care and poor nutritional status and lameness in donkeys have also been demonstrated; therefore, the importance of a healthy hoof is undeniable [1,5]. The veterinarian and farrier can have a significant influence on hoof health, gait, and general condition in a prophylactic, metaphylactic, and therapeutic manner [6,7]. However, this requires an adequate assessment of the hoof capsule and the bony and soft tissue structures it contains to assess which condition is considered normal or desirable and from which deviations a pathological condition can be deduced and must therefore be optimized. In most cases, clinical evaluation of the hooves can provide clues to the presence of such a pathological condition, but only the visualization of the hoof on radiographs permits accurate assessment of the interrelationships of external to internal structures and the objectification and quantification of the severity of pathological changes using morphometric measurements. The classic example of the practical use of this method is laminitis, in which measurements can be used to verify the rotation and sinking of the coffin bone. Other examples include the detection of keratomas, axial and conformational deviations, and imbalances. For the correct assessment of such conditions, many horse studies have resulted in reference values based on different length and angular measurements on projections of the toe. These findings serve as a guideline in daily work in which pathological change can be derived from deviation from the morphometric measured values. It has also been shown that the measured values significantly depend on factors, including horse breed, hoof trimming, and within an individual, between the right and left side and forelimb to hind limb [8,9,10,11]. As there are anatomical differences between donkeys and horses that cannot be ignored, especially in the hoof area, such as the U-shaped sole, the strongly developed frog, the thicker dorsal hoof wall and sole, the more distally located coffin bone within the hoof capsule, and the cubic appearance of the hoof [3,12,13,14,15,16], it can be assumed that the reference values determined for the horse cannot be transferred to the donkey without restriction [17]. A few authors have already determined measured values by radiologically examining front hooves of clinically sound donkeys and donkeys with specific hoof diseases or conformational abnormalities [18,19,20,21,22,23,24]. While the existing pool of corresponding data on the hindlimb is already considerably smaller in horses [25], to the author’s current knowledge there is no published study that has carried out morphometric measurements on the toe of the hindlimb of donkeys on radiographs.

The aim of this study is therefore to describe the physiological radiographic anatomy of the distal hind limb of domesticated, generally sound, and lameness-free European donkeys by generating mean values and standard deviations using length and angular measurements on the lateromedial and dorsoplantar radiographs of the right and left toes of the hind limb of donkeys. These should serve as a reference in practical work with donkeys in order to maintain hoof health and to objectify and simplify the detection of pathological conditions. In addition, (1) the influence of the projection-related magnification factor on the measurements should be investigated, (2) significant differences in the measurements between the right and left hind limb should be analyzed, and (3) correlations with various factors such as age, weight, and size should be determined.

## 2. Materials and Methods

### 2.1. Participating Subjects

Forty-six donkeys were included in the observational study between July 2022 and February 2024 that had to meet the following criteria: generally sound, no presently detectable apparent lameness in walk and no history of lameness according to the owners, and no influence of analgesic/antiphlogistic medication. In addition, hoof trimming had to be necessary due to the current condition of the hooves. At the beginning, available information on the medical history, usage, husbandry, and routine hoof management was collected after the owners had been informed and had given their consent. Due to the indication of optimizing routine hoof treatment, an ethical review and approval by the responsible animal welfare authority was not mandatory. The study was approved by the doctoral office of the Department of Veterinary Medicine at Justus Liebig University Giessen.

### 2.2. Procedure

The hooves were then trimmed and cleaned by approved farriers from the farriery of the Clinic for Equine Surgery and Orthopedics. Afterwards, radiopaque markers were applied to the hind hooves to highlight relevant measurement points on the radiographs. The distal limbs were imaged in the lateromedial and dorsoplantar projections according to a standardized X-ray procedure [26]. For the 90° image, the dorsal hoof wall was fitted with a wire, the proximal end of which was intended to mark the coronet band at the border to the hairy skin and ran to the ground directly along the dorsal hoof wall. The coronet band at the most plantar point was marked with a metal ball measuring 0.5 cm × 0.5 cm × 0.5 cm in the imaginary extension of the bale furrow to the plantar side at the transition to the hairy skin. A paper clip aligned vertically to the weight-bearing border was attached to the tip of the frog to mark the sole. For the 0° projection, the paper clip was left in position and the metal ball was removed, as was the wire on the dorsal hoof wall. Instead, two wires were fixed to the lateral and medial hoof wall at the widest point with the proximal end at the level of the coronet band at the transition to the hairy skin, which were orientated parallel to the course of the hoof horn in the direction of the weight-bearing border.

**Figure 1:**
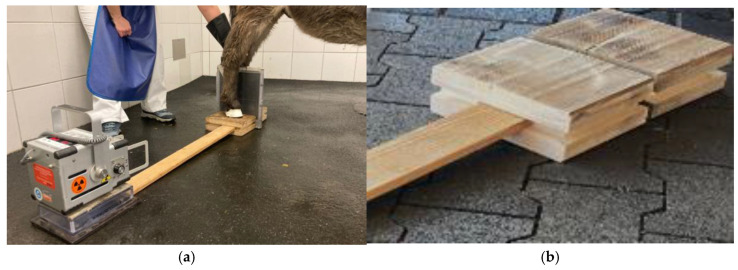
(**a**) The photo shows an example of a l.m. X-ray of the hoof on the right hind limb of a donkey. Both limbs are positioned on wooden blocks with equal weight-bearing. Using the inserted 80 cm long wooden slat, the central beam is positioned on the middle between the dorsal hoof wall and the heels orthogonally to the limb axis and the cassette. This X-ray image, for example, was taken with an X-ray device from Gierth^®^, Riesa, Germany. (**b**) The slat is inserted into the milled edge in the wooden block.

Both hind limbs were positioned on customized wooden blocks measuring 23 cm × 18 cm × 18 cm, with equal weight bearing. A metal ball measuring 0.5 × 0.5 × 0.5 cm was drilled into the center of the blocks, and a 2 cm deep edge was milled into each of the outer edges, into which an 80 cm long wooden slat was inserted to ensure correct alignment of the central beam and a repeatable equal distance between the X-ray unit and the cassette (Figure 1). As the X-ray images were generated with different portable X-ray systems due to the circumstances, the exposure settings (kV and mAS) were adapted individually for optimal imaging but were generally based on the settings recommended for the system, the animal species, and the anatomical localization.

The orientation of the central beam was chosen according to the standardized procedure described by Kummer et al. [26]. In detail, the central beam for the lateromedial image was focused on the middle between the dorsal hoof wall and the most plantar point of the coronary band, approx. 2 cm distal to the coronary band, parallel to the ground and orthogonal to the toe alignment and cassette. The cassette was positioned as close as possible to the limb at the medial edge of the block in accordance with the alignment of the central beam and the limb. In the dorsoplantar projection, the beam was centered on the dorsal hoof wall between the lateral and medial hoof wall and approx. 2 cm distal to the coronary band. The placement of the cassette was analogous to the criteria used for the lateromedial radiograph.

### 2.3. Measurements

The radiographs were transferred as DICOM formats to the data processing software EasyVET^®^, version number 10.20.7.4. The measurements were taken using the image processing tool contained in the software (EasyImage, VetZ, Isernhagen, Germany, https://easyimage.myvetsxl.com/).

**Figure 2:**
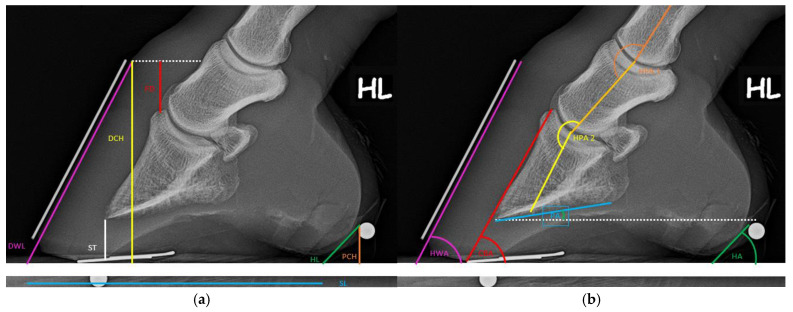
Length (**a**) and angular (**b**) measurements on the lateromedial radiograph.

Seven angular measurements and calculations, as well as seven length measurements, were carried out on the right and left sides of the lateromedial image (Figure 2; Table 1 and Table 2).

**Figure 3:**
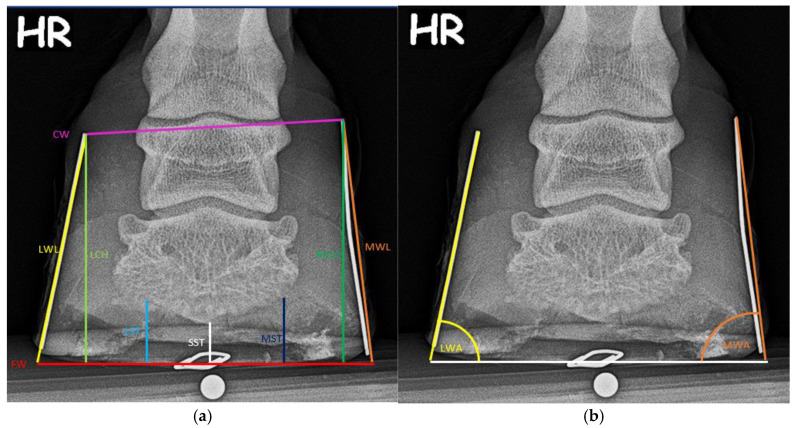
Length (**a**) and angular (**b**) measurements on the dorsoplantar radiograph.

Nine distances and two angles were obtained on the dorsoplantar projection. Abbreviations and definitions of the respective variables are listed in Table 3 and Table 4. The application of the various measurements is shown in Figure 3.

For the length measurements, the magnification effect caused by the X-ray was determined using the metal balls, which have the same known dimensions of 0.5 cm × 0.5 cm × 0.5 cm in each projection, and the absolute measured values were calculated as normalized values taking into account the respective individual magnification factor (formula: actual length = length measured radiographically × actual length of the marker/length of the marker measured radiographically [10]).

### 2.4. Statistical Analysis

For the statistical analysis using the SAS^®^ 9.4 software, the donkeys were divided into different groups according to the factors investigated: age, size, and weight. For age, a distinction was made between juvenile (0–4 years) and adult (>4 years). The size was categorized into small (≤105 cm height at the withers), medium (105–135 cm height at the withers), and large (≥135 cm height at the withers), mainly according to breed and weight [16]. Descriptive statistics with mean values, standard deviation, median, minimum, maximum, and 95% confidence interval limits were calculated for the distribution of breed, age, weight, sex and size, as well as for the individual variables. Due to the sometimes wide standard deviation of the data from the entire donkey population examined and the consequently poor applicability as reference values, the measurements of the group of adult, medium-sized domestic donkeys (GAMD) from the entire test population were also analyzed separately for each question in the further evaluations. Due to individual uncooperativeness of some subjects in some X-ray projections, the sample numbers for the affected variables are reduced accordingly.

A Spearman rank correlation was calculated for the question of whether the absolute measured values differ from the normalized values, and a Passing–Bablok regression was calculated after checking the linearity required for this using the CUSUM test. In order to analyze the variables for significant differences between the right and left hind limb, a paired *t*-test was carried out after the differences in the values were checked for normal distribution using the Shapiro–Wilk test. The distribution was also visualized graphically. To determine correlations between the hoof parameters and the variables of age, weight, and height of the test subjects, a Spearman rank correlation was calculated, and a graphical representation using a scatterplot was selected. When calculating the correlation regarding age and size, only the data from the entire donkey population was analyzed, as the specific donkey group (GAMD) only consists of adult, medium-sized individuals. The significance level for all calculations was set at *p* = 0.05.

### 2.5. Draft Preparation

The DeepL translator was used to optimize the wording and grammar when writing the original draft.

## 3. Results

### 3.1. Descriptive Analysis

Of the 46 donkeys kept in Germany that were included in the study during the period, lateromedial radiographs of the hind limbs were successfully obtained in a maximum of 41 donkeys due to individual lack of cooperation. Of these, 92.68% were adults (*n* = 38). The donkeys were categorized into three different groups, in which more than half (56.10%, *n* = 23) fell into the “medium” category. Five donkeys (12.20%), mainly the poitous (*n* = 4), were categorized as “large” and the remaining 13 donkeys, mainly represented by the dwarf donkeys, were categorized as “small” (31.71%). The average age in this population was 12.58 years, with the youngest animal being one year old and the eldest 40 years old. The donkeys weighed on average 191 kg, with a range of 90 kg (min.) to 409 kg (max.). There were 18 (43.90%) males and 23 (56.10%) females included.

In 37 right and 35 left hind limbs, a radiograph was successfully generated in the dorsoplantar projection. Also, in this population the domestic donkeys are the mainly represented with 86.49% (*n* = 32). The average age (12.78 y.) and the average weight (193.86 kg) are roughly comparable to the data of the population in which lateromedial radiographs could be taken, as is the gender distribution with 21 females and 16 males.

In the group of adult medium-sized domestic donkeys (GAMD), the sample size of the maximum X-ray images taken per hind limb is reduced to 21 right and 21 left feet in the lateromedial (l.m.) projection and 17 (left) and 19 (right) hooves in the dorsoplantar (d.p.) projection. The average age shifts by approximately one year to 14.19 years (l.m.) and 14.83 years (d.p.) compared to the total population, while the average weight of 189 kg (l.m.) and 193.86 kg (d.p.) is not subject to any relevant changes, but the range is noticeably narrower at 151 kg (min.) to 266 kg (max.). Regarding gender distribution, a clear shift in the male-to-female ratio of approx. 37% to 64% can be observed.

Both age and weight are not normally distributed in the populations analyzed.

The descriptive distributions mentioned above refer exclusively to the maximum available data on the two X-ray projections, which were calculated using the variables DWL for the l.m. projection and CW for the d.p. projection as examples. In individual cases, measurements for specific parameters are missing, so that the analyzed sample size and consequently the distribution of the analyzed factors (age, weight, etc.) shift individually for each variable. The specific sample size for each variable in the respective analyses is shown as “*n*” in the tables below.

All analyzed length and angular measurements on both the lateromedial and dorsoplantar X-ray projections are listed separately for the right and left toe of the hind limb in Table A3 and Table A5 (total population) and Table A4 and Table A6 (GAMD). Briefly, the dorsal hoof wall has an absolute length of approximately 70 to 80 mm and an angulation of 55° on the lateromedial projection; the coffin bone is approximately parallel to the hoof wall. The average founder distance is about 17 mm, and the sole is on average 14 mm thick. The heel angle averages 52°, which is somewhat flatter than the hoof wall angle. The HPA is broken forward in many cases and shows a very wide standard deviation. The PA is positive in the present populations. On the dorsoplantar projection, the measurements are characterized by the cubic appearance of the hoof, with outer hoof wall angles of almost 90° and hardly any discrepancies between the lateral wall lengths and the coronary band heights, as well as between the coronet width and the foot width.

### 3.2. Differences Between Measured Values and Normalized Values

In a comparison of the absolute length measurements of the variables determined on the lateromedial radiographs with the normalized values (DWL, ST, DCH, HL, PCH, FD), all variables show a significant, very strong positive correlation and a linear interrelationship, both in the overall population and in the group of adult medium-sized domestic donkeys (GAMD). No systematic and/or proportional differences were found (Appendix A Table A1 and Table A2). As a result, the following analyses focused primarily on the absolute measurements.

### 3.3. Differences Between the Right and Left Hind Limb

As shown in Table 5, the variables HL (*p* = 0.0051/0.002), PCH (*p* = 0.0084/0.009), and CW (*p* = 0.0361/0.03) show significant differences between the right and left hind limb in both the total population and in the GAMD. Compared to the left side, the right hind hoof of the total population of donkeys has on average 1.91 mm longer heels and correspondingly an average 1.42 mm longer plantar coronet band height. Right hooves on the dorsoplantar radiograph tend to have a narrower coronary band width compared to the left hind hoof, with an average difference of 0.77 mm (all).

### 3.4. Correlations

#### 3.4.1. Age

Regarding the age of the test subjects, no or only weak, non-significant dependencies of the variables can be proven. The only exceptions in the total population are the coffin bone angle (CBA) with rs = 0.28 and *p* = 0.02 and the axial deviation in the proximal interphalangeal joint (HPA1) with rs = −0.33 and *p* = 0.003.

#### 3.4.2. Weight

Overall, almost all length measurements in the total population show moderate to strong positive correlations with height and weight, whereas stronger correlations between the variables and weight can be observed (Table 6). With correlation coefficients of 0.54 (all)/0.59 (GAMD), 0.5 (all)/0.53 (GAMD), and 0.55 (all)/0.50 (GAMD), the dorsal coronet height (DCH), sole length (SL), and foot width (FW) show strong, significant (*p* < 0.05 in each case) correlations with weight in both populations studied. Further significant correlations were found between weight and the variables DWL, FD, CBA, and CW in both populations, with the dependency in the GAMD appearing to be moderate, while the latter variable showed a strong correlation in the total population. Sole thickness (ST) is the length measurement with the weakest correlation to the three factors analyzed.

#### 3.4.3. Height

The variables that correlate with height have lower correlation coefficients than the same correlating variables with weight. Consequently, the correlations observed are rather moderate overall (Table 6).

#### 3.4.4. Angular Measurements

Overall, rather weak to moderate correlations can be found in the angular measurements compared to the length measurements. However, the strongest correlations are also found with weight, followed by height and finally age. The angular parameters CR, HA, MWA, and LWA show no correlations with the analyzed factors age, weight, and height. The axial deviations in the proximal and distal interphalangeal joints (HPA1 and 2) are the only variables analyzed to show significant, negative correlations with weight and height (Table 6).

## 4. Discussion

The aim of this study was to describe the physiological radiographic anatomy of the distal hind limb of domesticated, generally sound, and lameness-free European donkeys using morphometric measurements. As shown in Appendix A, Table A3, Table A4, Table A5 and Table A6 the descriptive data of the entire donkey population (n max. = 41) of all ages, sizes, phenotypes, and weights show approximately two to three times wider standard deviations of the mean values for some length measurements when compared with those of the group of adult domestic donkeys of medium size (GAMD). The mean values, on the other hand, show noticeably smaller differences between the entire donkey population and the GAMD, although the values are generally slightly smaller in the GAMD. In contrast, the angular measurements are almost identical between the two populations. The variables describing the distance from the coffin bone to the ground (ST, SST, LST, MST) are the length measurements with the smallest divergence between the values of the total population and the selected donkey group. The smaller dispersion of the standard deviation in the GAMD reflects the smaller variation in size within this population and, conversely, also explains why the distribution in the population, which includes all breeds and sizes, is significantly wider. As angles are independent of length measurements, it is not surprising that these are also very similar between the two populations and can therefore be used to objectively assess hoof conditions, regardless of breed or size. This is also confirmed by the correlation calculations of the variables for age, size, and weight, which are shown in Table 6. Here, too, the angular measurements show only rather weak to moderate and few significant correlations with age, weight, and height. It is also noticeable here that the sole thickness parameters show the weakest correlations between length measurements and height, age, and weight.

Consequently, it can be deduced for daily use that the sole thickness and angular measurements are particularly suitable for comparing the measured values collected in individual cases with the data published here, as these appear to be more independent of age, weight, and height. Furthermore, no relevant influence of age on the data can be determined here, so that this can be regarded as negligible for the evaluation of radiographs of the distal toe of hind limbs of donkeys anyway. Wacker et al. came to comparable results when they investigated radiographic coffin bone changes in a donkey population regarding the influence of age [17]. In 2006, Kummer et al. were also unsuccessful in establishing a correlation between the parameters measured on radiographs of warmbloods and age [9]. Only in the first year of life was a significant correlation of age with the change in morphometric measurements of the toe of horses proven [27]. In addition, a significant relation between the severity of lameness and increasing age was found in highly prevalent lame draft donkeys in Pakistan [5], as well as a significant correlation between the presence of lameness and increasing age in another Pakistani working donkey population [28]. Due to the inclusion criteria, which require general soundness and absence of lameness in walk, lame, presumably older, donkeys were not examined radiographically. Lameness can be an indirect indication of a morphometric deviation, so that it can be presumed that a correlation of morphometric parameters with age is potentially considered possible if lame and therefore very likely older donkeys are included in the study population. Consequently, a correlation cannot be proven based on the data available here, but it should nevertheless be taken into consideration. Further studies are required to determine more precisely the influence of age on the measurement variables and a potential causal relationship with a pathological condition.

In contrast, almost all remaining length measurements show moderate to strong, significant correlations with weight and size in the overall population; the strongest correlations are observed for dorsal coronet height (DCH), sole length (SL), and foot width (FW). In particular, foot width (FW) is also reported in the related literature to be strongly and positively correlated with height and weight in both donkeys and horses [10,29,30]. Additionally, similar conclusions as in the present study can be found that primarily the length measurements correlate with weight and height [9,11], while the angular measurements and the parameters related to sole thickness (ST, SST, MST, and LST) show rather weak and partially non-significant correlations [11]. In other studies in horses, no significant changes in founder distance (FD) could be determined in connection with height or weight [10,26], while the data available here for the total population and GAMD (*) suggest a moderate, significant correlation with weight (rs = 0.47/0.41 *, *p* < 0.0001/ = 0.008 *) and height (rs = 0.35, *p* = 0.002), as was also observed in a conformation study in ponies [11]. An influence of the factors on the measured sinking of the coffin bone within the hoof capsule can therefore not be excluded.

Furthermore, as a result of comparative studies of morphometric measurements intra vitam and postmortem in both donkeys and horses, it is assumed that the load on the limb also has a relevant influence on the measurement of the founder distance [26,31]. Multiple studies prove that the founder distance should not be compared between donkeys and horses in general due to the different hoof anatomy, especially the more distal coffin bone in donkeys [2,3,14,18,32,33]. Considering this, the author agrees with other authors in the opinion that the founder distance and the subsequent deduction of a pathological condition of the hoof in the form of laminitis should be interpreted with the highest degree of caution, as the differentiation between normal and deviation from normal findings is very sensitive to error due to the inevitable dependence on factors such as species, breed, weight, size, load, and forelimb to hind limb, particularly in the case of mild changes. In addition, the author agrees that a multi-parameter approach in combination with anamnestic indications and clinical findings is the most reliable method of verifying a pathological condition in the hoof [11,18,19,20,26,34,35]. As the results presented here also suggest, Lesniak et al. demonstrated a stronger influence of weight on hoof conformation in horses compared to size in their study [29]. Wacker et al. also identified a significant, positive correlation between the weight of the donkeys and the coffin bone changes [17].

In the studies dealing with the morphometric normal anatomy of the donkey’s toe, it was deduced and hypothesized from the data that the measured parameters are subject to a strong breed-related influence and that it is therefore advisable to generate and provide specific, validated morphometric reference values for each breed [18,19,30,36]. This has already been proven in the horse population by multiple conformation studies of different horse breeds [9,10,11,25,37,38]. The strong divergence of the standard deviations between the total population and the population of medium-sized adult domestic donkeys in the current study, as well as evidence of some strong dependencies on weight and size, which are also linked to a specific breed exterior, support this recommendation in order to differentiate normal findings from pathological findings as accurately as possible. Consequently, the influence of weight, size, and breed should be considered when evaluating for pathological changes, particularly in the case of linear measurement variables.

The influence of the projection-related magnification of the measured values on the X-ray images must also be considered. A very strong, positive, significant correlation with a linear relationship between the absolute measured values and the normalized, corrected values was proven here, so that the magnification factor can be considered negligible. From a scientific point of view, it is unquestionable that the use of the standardized values is more correct and precise, as they reflect the real measured distance and can also guarantee the most reliable comparability in repeat examinations. Since the realization in field conditions is rather impractical, the authors consider the conversion to be non-mandatory based on slight deviations. If the differentiation between pathological and normal findings based on the measurements is unclear in a questionable case, the calculation of the artificial magnification can nevertheless prove helpful.

In this study, only three variables (HL, PCH, and CW) showed significant differences between the right and left hind limb. A significant difference was also calculated for sole length (SL) in the total population, but this significance is not permissible due to the non-normally distributed values and is also not confirmed in the normally distributed GAMD, so that this statistical anomaly is to be considered irrelevant. Compared to the left side, the right hind hoof tended to have longer heels and a correspondingly higher plantar coronet band height. Due to the slight difference in the average width of the coronet band between the right and left side, the discrepancy between the two hind limbs is considered negligible despite the calculated statistical significance. Comparably, right–left asymmetries were found in morphometric examinations of working donkeys in Pakistan, in which the right front hoof also showed statistically significant (*p* = 0.02) longer heels with a difference of 0.28 cm compared to the left, in addition to a longer medial hoof wall (MWL) and a longer sole [28]. Kummer et al. showed in morphometric studies on the hooves of warmbloods that the right front hoof had a longer dorsal hoof wall (DWL) in 70% of cases, while the bony structures were larger on the left side on both the LM and DP projections [9]. A side preference of horses has been discussed as an explanation, as has already been investigated in Thoroughbreds, for example [39]. Thieme et al. found rather minor differences between the right and left limbs of the pony population studied, but they also noted an asymmetry on the LM image, in which both the front and hind limbs had significantly larger left hooves (FL) and the left hooves tended to be flatter, while the right, smaller hooves were more steeply angled and also deduced side preferences of the horses from this [11]. Lesniak et al. also observed an increasing asymmetry between the right and left forelimb with growing mass and size; the left forelimb tended to have a more acute angle, i.e., to become flatter, while the right forelimb appeared steep and cubic [29]. In the population studied by Cripps and Eustace, no significant differences between the right and left forelimb could be detected [10], nor in the hind limbs of the horses studied by Kalka et al. [25] and in the front hooves of the Amiata donkeys [19]. Although no significant divergence of the DWL or DWA and thus no evidence of massive hoof asymmetry between the right and left limb, as described in horses, can be found in the data presented here, there is accumulating evidence that a longer heel on the right hoof is a more frequently observed morphometric hoof asymmetry in horses and donkeys. Presumably this is also due to individual laterality and the resulting load and growth asymmetries, but otherwise no relevant differences between the left and right hind limb were found in this study, so that the existence as well as the relevance of a possible laterality regarding morphometric measurements on the hind limbs of donkeys should be viewed critically and require further investigation.

As only the measurements of the hind limb were evaluated in this study, no statistical analysis of the comparison of the data between the forelimb and hind limb is available. Table 7 and Table 8 compare the measured absolute mean values and standard deviations of the entire donkey population (all), the selected group of adult domestic donkeys of medium size (GAMD), and analogously collected parameters in other studies of horses and donkeys on the front and hind toes, so that the values are compared and discussed below.

Basically, there is only one other publication that provides morphometric measurement data on the hind hooves of donkeys [30], so that the comparison is otherwise made with the also rare baseline data collected on hind limbs of different horse breeds [10,11,25,37,38,40,41]. For the front hooves, there is significantly more published data for donkeys of different breeds, use, and origin [18,19,20,21,24,28,30], so that the comparison here focuses on the donkeys and the horses are excluded.

**Table 7:** Comparison with literature, compared with other hindlimbs.

v	Schaus et al. (Present Study)				Mostafa et al. 2020 [30]		Other Authors		
	All		GAMD		Donkeys **		Horses and Ponies		
	Right Hind	Left Hind	Right Hind	Left Hind	Right Hind	Left Hind	Right Hind	Left Hind	Publication
DWL ^1^	83.93 ± 21.51	83.56 ± 20.62	79.25 ± 10.00	77.82 ± 7.67	68.4 ± 3.60		80.38 ± 11.15	80.53 ± 10.94	[11]
							78.00 ± 11.00		[41]
								86.00 ± 6.00	[37]
ST ^1^	15.57 ± 4.30	15.16 ± 4.09	15.78 ± 3.60	14.12 ± 3.23			10.37 ± 2.40	10.31 ± 2.14	[11]
							20.00 (˜x)		[38]
DCH ^1^	70.80 ± 18.65	70.04 ± 18.45	67.90 ± 9.59	65.99 ± 8.24				69.00 ± 4.00	[37]
HL ^1^	17.96 ± 5.72	16.05 ± 4.07	18.39 ± 6.04	15.58 ± 4.06	33.90 ± 3.30 *	34.10 ±3.60 ’	43.00 ± 12.00 *	43.00 ± 10.00 ’	[41]
PCH ^1^	14.38 ± 4.87	12.96 ± 3.61	14.41 ± 5.19	12.32 ± 3.81					
FD ^1^	18.77 ± 7.31	18.02 ± 7.73	17.46 ± 3.73	17.19 ± 3.65			8.77 ± 2.35	8.72 ± 2.45	[11]
							11.90 (˜x)		[38]
							5.22 ± 2.79		[10]
SL ^1^	114.36 ± 32.20	117.47 ± 30.85	106.36 ± 14.33	108.38 ± 13.43			121.51 ± 18.97	124.54 ± 19.52	[11]
							117.00 ± 17.00		[41]
								114.00 ± 8.00	[37]
HWA ^2^	55.72 ± 4.31	55.39 ± 4.19	56.77 ± 4.82	56.46 ± 3.68	59.61 ± 3.48		50.86 ± 3.54	50.95 ± 3.24	[11]
							53.70 ± 5.70		[41]
							48.32 (˜x)		[38]
							50.90 ± 3.70	50.90 ± 3.60	[25]
								52.00 ± 4.00	[37]
							49.08 ± 3.18		[10]
CBA ^2^	56.67 ± 4.70	55.80 ± 4.43	58.15 ± 4.62	56.95 ± 4.13	49.5 (˜x)	50.4 (˜x)	50.86 ± 3.54	50.95 ± 3.24	[11]
							49.09 (˜x)		[38]
							48.94 ± 3.06		[10]
CR_c ^2^	0.95 ± 3.29	0.42 ± 3.01	1.38 ± 3.58	0.49 ± 2.90			49.75 ± 3.85	49.98 ± 3.49	[11]
							−0.14 ± 1.59		[10]
CR_m ^2^	1.26 ± 3.27	1.32 ± 2.92	1.70 ± 3.39	0.87 ± 3.01			−1.1 ± 1.41	−0.97 ± 1.46	[11]
PA ^2^	6.21 ± 4.02	5.24 ± 4.09	6.80 ± 3.00	5.64 ± 3.80			−2.75 ± 2.80	−4.00 ± 2.30	[40]
							2.81 (˜x)		[38]
								4.00 ± 7.00	[37]
HA ^2^	52.81 ± 5.81	53.01 ± 6.04	52.17 ± 5.89	52.27 ± 6.95	56.89 ± 3.64		70.40 ± 6.10 *	75.10 ± 6.00 ’	[41]
							36.60 ± 9.70	36.30 ± 9.40	[25]
HPA1 ^2^	5.4 ± 8.82	4.34 ± 9.47	3.62 ± 8.84	2.04 ± 10.48			−10.50 ± 3.10	−9.90 ± 2.70	[40]
HPA2 ^2^	−0.67 ± 11.08	−3.14 ± 10.11	−4.34 ± 12.69	−6.14 ± 8.70	−13.10 ± 9.86		−13.90 ± 4.50	−16.5 ± 5.60	[40]
							6.64 ± 25.64		[10]
LWA ^2^	88.23 ± 4.55	87.22 ± 4.41	87.24 ± 4.50	86.63 ± 4.35				76.00 ± 4.00	[37]
MWA ^2^	88.84 ± 4.54	88.64 ± 4.10	87.72 ± 3.76	88.04 ± 3.58				81.00 ± 4.00	[37]
CW ^1^	80.33 ± 24.95	81.10 ± 26.03	73.93 ± 11.39	73.77 ± 8.68					
FW ^1^	83.37 ± 23.04	83.65 ± 23.67	78.23 ± 11.35	78.07 ± 9.40	63.30 ± 3.70		100 ± 18.00		[41]
								108.00 ± 6.00	[37]
LWL ^1^	61.39 ± 17.67	62.26 ± 16.53	58.38 ± 5.63	58.67 ± 6.58					
MWL ^1^	59.52 ± 16.87	61.02 ± 19.19	57.37 ± 6.52	56.21 ± 7.65					
LCH ^1^	60.93 ± 17.55	61.94 ± 17.12	58.02 ± 5.42	57.84 ± 6.63					
MCH ^1^	59.56 ± 17.18	59.85 ± 18.64	57.92 ± 9.05	54.91 ± 7.26					
SST ^1^	16.71 ± 6.00	16.53 ± 4.61	16.29 ± 4.20	16.10 ± 3.13					
LST ^1^	20.36 ± 7.22	19.53 ± 5.88	19.73 ± 4.53	18.88 ± 3.57					
MST ^1^	19.80 ± 6. 12	20.02 ± 9.83	19.58 ± 4.02	21.40 ± 12.19					

^1^ = in mm, ^2^ = in °, ˜x = median, * = lateral, ’ = medial, v = variable, ** = working donkeys from Egypt, GAMD = group of adult medium-sized donkeys, [11] = Thieme 2015, [41] = Mellish 2023, [37] = Herbrecht 2020, [38] = Cardona 2021, [10] = Cripps and Eustace 1999, [25] = Kalka 2021, [40] = Sharp 2022, data for right/left are shown in the specified column. In cases where no specification was made, the data are centered between both columns.

**Table 8:** Comparison with literature, compared with front limbs of donkeys.

v	Schaus et al. (Present Study)				Wacker [24]		Other Authors		
	All		GAMD		All		Donkeys		
	Right Hind	Left Hind	Right Hind	Left Hind	Right Front	Left Front	Right Front	Left Front	Publication
DWL ^1^	83.93 ± 21.51	83.56 ± 20.62	79.25 ± 10.00	77.82 ± 7.67	81.41 ± 23.00	80.41 ± 22.07	78.33 ± 8.60		[21]
							66.60 ± 9.20	65.70 ± 10.10	[28]
							80.00 ± 2.70		[19]
							77.40 ± 0.2		[20]
ST ^1^	15.57 ± 4.30	15.16 ± 4.09	15.78 ± 3.60	14.12 ± 3.23	16.66 ± 5.83	15.82 ± 4.50	23.00 ± 4.34		[21]
							18.70 ± 1.10		[19]
							24.30 ± 0.2		[20]
DCH ^1^	70.80 ± 18.65	70.04 ± 18.45	67.90 ± 9.59	65.99 ± 8.24	70.03 ± 19.82	69.16 ± 19.11	68.47 ± 8.96		[21]
HL ^1^	17.96 ± 5.72	16.05 ± 4.07	18.39 ± 6.04	15.58 ± 4.06	18.48 ± 5.65	20.29 ± 5.95	47.13 ± 10.24		[21]
							34.90 ± 9.10	32.20 ± 7.40	[28]
PCH ^1^	14.38 ± 4.87	12.96 ± 3.61	14.41 ± 5.19	12.32 ± 3.81	14.92 ± 4.89	16.28 ± 5.81	31.70 ± 6.08		[21]
FD1	18.77 ± 7.31	18.02 ± 7.73	17.46 ± 3.73	17.19 ± 3.65	18.39 ± 7.52	18.50 ± 7.31	19.0 ± 0.80		[19]
							25.20 ± 31.80		[20]
								10.40 ± 3.66	[18]
SL1	114.36 ± 32.20	117.47 ± 30.85	106.36 ± 14.33	108.38 ± 13.43			86.57 ± 9.47		[21]
							89.60 ± 13.10	87.50 ± 11.50	[28]
							127.50 ± 3.40		[19]
							95.40 ± 0.20		[20]
HWA ^2^	55.72 ± 4.31	55.39 ± 4.19	56.77 ± 4.82	56.46 ± 3.68	58.28 ± 4.63	57.97 ± 4.68	58.60 ± 4.39		[21]
							62.52 ± 8.29	61.48 ± 8.17	[28]
							57.30 ± 0.7		[19]
							70.2 ± 88.5		[20]
								61.61 ± 5.24	[18]
CBA ^2^	56.67 ± 4.70	55.80 ± 4.43	58.15 ± 4.62	56.95 ± 4.13	59.57 ± 4.62	59.14 ± 4.47	57.00 ± 4.30		[21]
							58.00 ± 1.00		[19]
							70.2 ± 95.4		[20]
								64.11 ± 4.70	[18]
CR_c ^2^	0.95 ± 3.29	0.42 ± 3.01	1.38 ± 3.58	0.49 ± 2.90	1.29 ± 3.65	1.17 ± 3.50	1.40 ± 0.80		[19]
								2.50 ± 3.07	[18]
CR_m ^2^	1.26 ± 3.27	1.32 ± 2.92	1.70 ± 3.39	0.87 ± 3.01	1.45 ± 3.73	1.27 ± 3.73			
PA ^2^	6.21 ± 4.02	5.24 ± 4.09	6.80 ± 3.00	5.64 ± 3.80	6.37 ± 3.98	6.09 ± 3.23	4.80 ± 1.64		[21]
							25.0 ± 31.6		[20]
								8.26 ± 4.75	[18]
HA ^2^	52.81 ± 5.81	53.01 ± 6.04	52.17 ± 5.89	52.27 ± 6.95	50.58 ± 8.82	51.59 ± 8.82	53.20 ± 7.98		[21]
							54.89 ± 8.33	56.39 ± 9.34	[28]
HPA1 ^2^	5.4 ± 8.82	4.34 ± 9.47	3.62 ± 8.84	2.04 ± 10.48	9.27 ± 4.64	9.77 ± 4.01	9.0 ± 0.9		[19]
								5.11 ± 5.20	[18]
HPA2 ^2^	−0.67 ± 11.08	−3.14 ± 10.11	−4.34± 12.69	−6.14 ± 8.70	−9.71 ± 10.26	−8.96 ± 8.71	2.50 ± 12.24		[21]
							−13.48 ± 10.48	−11.52 ± 8.51	[28]
							8.1 ± 1.1 *		[19]
								−4.30 ± 10.40	[18]
LWA ^2^	88.23 ± 4.55	87.22 ± 4.41	87.24 ± 4.50	86.63 ± 4.35	87.47 ± 4.68	86.87 ± 4.77	87.80 ± 2.05		[21]
							43.80 ± 44.70		[20]
MWA ^2^	88.84± 4.54	88.64± 4.10	87.72 ± 3.76	88.04 ± 3.58	88.63 ± 4.88	89.61 ± 5.72	89.4 ± 4.45		[21]
							20.20 ± 0.10		[20]
CW ^1^	80.33 ± 24.95	81.10 ± 26.03	73.93 ± 11.39	73.77 ± 8.68	84.62 ±23.87	83.84 ± 24.82			
FW ^1^	83.37 ± 23.04	83.65 ± 23.67	78.23 ± 11.35	78.07 ± 9.40	87.39 ± 22.36	86.43 ± 23.20	63.80 ± 8.40	62.20 ± 8.40	[28]
							100 ± 1.30 mm		[19]
							68.60 ± 0.4		[20]
LWL ^1^	61.39 ± 17.67	62.26 ± 16.53	58.38 ± 5.63	58.67 ± 6.58	62.96 ± 16.37	63.20 ± 15.50	57.60 ± 15.94		[21]
							54.90 ± 8.10	55.80 ± 9.00	[28]
							72.50 ± 1.10		[19]
							49.50 ± 0.40		[20]
MWL ^1^	59.52 ± 16.87	61.02 ± 19.19	57.37 ± 6.52	56.21 ± 7.65	61.76 ± 16.94	62.03 ± 15.61	60.47 ± 18.07		[21]
							59.90 ± 9.70	57.20 ± 8.50	[28]
							65.00 ± 1.20		[19]
							55.30 ± 0.20		[20]
LCH ^1^	60.93 ± 17.55	61.94 ± 17.12	58.02 ± 5.42	57.84 ± 6.63	62.42 ± 16.22	62.36 ± 15.45	57.47 ± 15.71		[21]
MCH ^1^	59.56 ± 17.18	59.85 ± 18.64	57.92 ± 9.05	54.91 ± 7.26	61.19 ± 16.96	61.57 ± 15.25	60.27 ± 17.97		[21]
SST ^1^	16.71 ± 6.00	16.53 ± 4.61	16.29 ± 4.20	16.10 ± 3.13	17.60 ± 5.30	17.24 ± 4.99			
LST ^1^	20.36 ± 7.22	19.53 ± 5.88	19.73 ± 4.53	18.88 ± 3.57	19.75 ± 5.73	19.39 ± 5.59	27.73 ± 5.95		[21]
MST ^1^	19.80 ± 6. 12	20.02 ± 9.83	19.58 ± 4.02	21.40 ± 12.19	18.23 ± 5.60	17.93 ± 4.89	27.00 ± 8.70		[21]

^1^ = in mm, ^2^ = in °, v = variable, GAMD = group of adult medium-sized donkeys, * angle between the long axis of the middle phalanx and the ground, [21] = El-Marakby 2024, [28] = Khan 2024, [19] = Nocera 2020, [20] = El-Shafaey 2017, [18] = Collins 2011, data for right/left are shown in the specified column. In cases where no specification was made, the data are centered between both columns.

### 4.1. Distance Measurements on the LM View

The distance measurements taken on the lateromedial radiograph (DWL, ST, DCH, HL, PCH, FD, SL) already show some differences. Our data suggest that the average dorsal hoof wall length of the hind limb in European donkeys is approximately between 70 and 75 mm (normalized), or approximately 80 mm without correction for artificial magnification. The Egyptian donkeys showed an average dorsal hoof wall length of 68.4 mm, which, compared to the data available here, was only subject to a significantly smaller variance [30] and thus showed slightly shorter dorsal hoof walls overall compared to the population studied here. In retrospect, the average measurements of the various smaller-framed horse breeds are close to the mean values found here. It is noteworthy that the Icelandic horses had approximately 10 mm longer dorsal hoof walls on the examined left hind hooves, despite their relatively similar stature [37]. In their study, Herbrecht et al. also demonstrated positive correlations between tölt performance and the length of the dorsal hoof wall, so it can be concluded that dorsal hoof walls are traditionally maintained longer in Icelandic horses during routine hoof trimming, regardless of the critical biomechanical additional load on the hoof postulated by the authors [37]. The data of the front hooves of the different donkey populations are in line with the average values of the dorsal hoof wall length found here. The only exception is the Pakistani draft donkeys studied by Khan et al. [28], which in turn show comparable average values to the Egyptian donkeys of Mostafa et al. [30]. It is remarkable that in these two studies, measurements were made based on digitalized photographs, whereas in the other studies they were derived from X-ray images. This observation may suggest an influence of the respective imaging method on the measurements, but comparative measurement accuracy studies between digitalized photographs and X-ray images show the equality and comparability of both techniques [42]. On the other hand, morphometric studies using photographs and radiographs have found differences in individual variables (e.g., DWA, HA), albeit minor but present [21,43,44]. Consequently, although slight differences can be expected when comparing the measured values of two different imaging methods, the detection of a pathological condition, taking into account influencing factors such as weight or size, should nevertheless be guaranteed based on massive deviations. Due to the proven influences of hoof trimming on morphometric measurements [8,9], this aspect should also be considered when comparing the published data. In Mostafa et al., for example, the donkey hooves were gently rasped for better comparability, but no complete routine hoof trimming was carried out [30], as was the case in this study.

The donkey population studied here has a quite homogeneous average sole thickness of approximately 13 to 15 mm, while ponies tended to have a distance of around 10 mm between the tip of the coffin bone and the sole surface [11]. The sole thickness of the hind hooves of Colombian Paso horses examined by Cardona et al. is similar to the values measured radiologically on the front hooves of donkeys and, at 18.7 mm to 24.3 mm, are considerably higher than the sole thicknesses measured here [38]. In connection with the laminitis-associated radiological changes in the form of lowering and rotation of the coffin bone, which also occur in donkeys [18,36], a reduced sole thickness may also be noticeable on the radiograph. However, the values measured here should not automatically be assumed to indicate laminitis just because they are lower than the data previously published for donkeys, as the classification for thin soles defines values < 10 mm as suspicious and <5 mm as very thin [45]. Rather, despite the apparently low dependence on external factors and the narrow standard deviation, this value should be understood as individual and should only be considered as a reason to suspect a pathological condition of the hoof if it falls below the critical limit values. It is possible that the time of the last hoof trimming and the individual style of the respective farrier in the various publications contributed to the divergences, as these factors have been shown to have a considerable influence on the measurements of hooves [8,26].

In the present donkey population, average heel lengths between 15 and 18 mm were measured, whereas other comparable literature found significantly longer heels in horses and donkeys with average values approximately twice as large or larger at both front and hind. Due to the sometimes irregular shape of the soft tissue shadow on radiographs, the measurement of heel length is not easy to perform in a standardized manner based on the author’s personal experience, so this is a potential source of measurement inaccuracy. Likewise, as with the individual assessment of each measurement variable, all potential influences should be considered, such as the last hoof trimming, for example. Although Kummer et al. [8,26] did not analyze the HL, it cannot be ruled out that the length of the heel is also affected due to the findings that hoof preparation has a considerable influence on the measured conformation. In studies on feral horses, long heels were found in the majority of cases, as well as correlations with the distances traveled and the ground conditions, showing that environmental factors can also influence this variable [41,46].

The founder distance was first described by Cripps and Eustace in 1999 and validated as a laminitis parameter; from their analyses, they concluded that FD on the hind limbs of healthy horses should not be >7 mm [10]. In 2011, Collins et al. investigated this variable in healthy European donkeys and donkeys with laminitis in a similar way and concluded from the data that a coffin bone sinking is suspected in donkeys from a founder distance >13 mm [18]. In this studied population of generally sound, lameness-free European donkeys, an average FD of around 16 to 18 mm was measured. The fact that this variable shows significantly larger measured values compared to the horses confirms the known lower position of the coffin bone in the hoof capsule [2,3,14,33,45]. Compared to the other donkey populations studied, the FD of the European donkeys studied here is greater than that determined by Collins et al. [18] for healthy donkey hooves but smaller than in the donkeys studied from Italy and Egypt [19,20]. A re-evaluation of the critical threshold at which a donkey’s coffin bone sinking can be diagnosed based on the founder distance should therefore be considered based on further investigations. Furthermore, as other authors have already emphasized, the founder distance should be interpreted with caution, as there are indications that it is influenced by factors such as weight and size [11], the load on the limb [26,32], the inadequate adjustment of the central beam [10], and is subject to intra- and inter-individual variations even in a healthy hoof condition [34].

In the donkey populations studied here, an average sole length (SL) of approximately 100 to 110 mm was measured, which shows a wide variation that can be plausibly explained by the strong positive correlation with weight and size that has been proven here as well as for warmbloods [9]. Due to the steeper hoof wall compared to horses [3,12,13,16,33], the sole length is generally shorter in donkeys than in horses. However, Table 7 shows that the average values for Canadian feral horses [41] and Icelandic horses [37] are similar to those presented here. These can be explained by the massive deformation of the hooves in the former case and the steeper hoof wall angles in the latter. In comparison to the examined front hooves of donkeys, the Pakistani and Egyptian working donkey populations show shorter sole and foot lengths of approx. 80 to 90 mm with significantly less spread [20,21,28], while the Amiata donkeys show significantly greater values with an average of approx. 130 mm [19]. Khan et al. were also able to prove significant differences in sole length between the right and left front toes with an average 0.22 cm longer right sole [28], while in a pony population studied, the left hind hooves of 66.7% had 3 mm longer soles on average, which are statistically significant [11]. In the current study, no relevant difference in this parameter between the right and left hind limb can be proven. In addition, conformational deviations in the form of long toes and underrun heels have a reducing influence on sole length [21]. Another factor that decreases sole length is hoof trimming, which shortens the sole length by an average of 5–6 mm in warmbloods [9]. When comparing this variable between different publications, it is important to consider discrepancies occurring in the definition of the measuring points of sole length, which is often referred to synonymously as foot length (FL) [34]. The hoof balance can be calculated based on the sole length and the perpendicular from the center of rotation of the coffin joint [34]; this parameter was not examined in the present study.

### 4.2. Angular Measurements on the Lateromedial View

As the average measurements of the hoof wall angle (HWA) and the coffin bone angle (CBA) are 55° to 56° and 56° to 58°, respectively, and the low measured and calculated coffin bone rotation (CR_m/c = CBA − HWA) are 0.4° to 1.7°, the values confirm the parallelism of the dorsal hoof wall to the dorsal surface of the coffin bone postulated in both healthy horse and donkey toes [2,18,34], as well as the 5° to 10° steeper dorsal hoof wall in donkeys compared to horses, as described by multiple authors [3,12,13,16,33]. Referring to Table 7, horses have an average hoof angle of around 50° on the hind limb, while donkeys have an angle of between 56° and up to around 60° [18,19,21,24,28,30] in both hind and front hooves. One study even measured average hoof angles of 70° [20]. A statistically validated comparison with the forelimb is not possible as only the hind hooves were analyzed in this database. However, a comparison of the literature on the front hooves of the donkeys studied does indicate that the hind hooves may be slightly flatter than the front hooves, as has also been shown in studies on horses [10,11,25]. This is contradicted by the fact that Cardona et al. in Paso horses and Mostafa et al. in Egyptian donkeys found no significant differences in the hoof wall angle between the forelimb and hind limb [30,38] as well as by reports of no significant differences between hind and front limbs in donkeys and mules **[13]**.

In the measurement data available here, the plantar angle is around 5 to 6° and is therefore similar to the basic data determined for the hind limbs of various horse breeds. Since, to the author’s knowledge, no reference values have been defined for the donkey and all values available for comparison are also at least 5° or more, a desirable positive plantar angle can be assumed, analogous to the horse. Studies in horses have shown that negative plantar angles are significantly associated with hindlimb lameness and altered posture of the hind limbs [40,47].

Based on the assumption for the horse that the heel angle should correspond to the hoof wall angle and consequently also to the coffin bone angle on the healthy hoof [34], the angles of 52° to 53° measured here show that the heels are not completely parallel to the hoof wall but are slightly flatter. This corresponds to the findings of comparable work on horses and donkeys, both on forelimbs and hind limbs [21,24,25,28,30,44]. In their studies on donkeys with normal hooves and donkeys with conformational deviations in the form of long toes and underrun heels, El-Marakby et al. observed a partial approximation of the heel angle to the hoof angle with the aforementioned conformational deviation [21], as well as demonstrating a positive correlation between the hoof wall angle and the heel angle [23]. Table 7 and Table 8 show that the HA is between 50 and 56° in all donkey populations studied to date and is significantly smaller in horses than in donkeys (36° to 37°), comparable to the hoof wall angle, as expected. Additionally, HA is slightly flatter than the corresponding hoof angle in most of the cases. The only exception is the feral horses from Sable Island, where the heel angle is about 20° greater than the hoof angle [41]. Furthermore, these animals showed long heels in 85% of cases and 100% conformational deviations [41], so that the normal condition of the hooves should not be deduced from this individual case due to influences such as conformational deviations affecting the dorsal hoof wall as well as the effect of the substrate and lack of trimming on hoof growth.

Some authors consider a mildly broken-forward hoof pastern axis to be a normal finding in donkeys [2,3,18] and is similarly observed in the donkey population studied here in the form of the axial deviation in the coffin joint (HPA2) as a negative value of −1° to −6°, as well as in most other data published on donkeys. The considerable variance of this measurement emphasized by Collins et al. [18] is also evident in the standard deviation values presented here and in the literature comparison. Broken toe axes are also found in the hind limbs of horses, where a greater deviation was also measured in the coffin joint than in the proximal interphalangeal joint. Furthermore, a correlation with the correction of a negative plantar angle by hoof trimming and the resulting change in posture was found [40]. Another pathological condition of the equine hoof associated with a broken-forward hoof pastern axis is the clubfoot. As Wacker et al. and Bartmann and Pietta concluded after transferring the criteria used to define a clubfoot for the horse to the donkey, hyperextension in the coffin joint in combination with an obtuse hoof angulation of at least 70° is not to be considered a physiological finding in the donkey either [13,16,17]. This argument is also supported by the findings that the hoof pastern axis in obviously lame donkeys is broken significantly more anteriorly than in lameness-free donkeys [28] and that donkeys more frequently show severe lameness in the hind limbs, where there is also a high prevalence of broken-forward hoof axes in combination with painful reactions on palpation [5]. Although the average of the donkeys examined in this publication suggests that many animals have a hoof pastern axis that is broken forward to a minor degree, there are also individuals that have a physiological, stretched toe axis. Hoof trimming should be based on the toe axis, and, if necessary, the heels should be shortened to avoid a toe axis that is broken forward [3,13,33].

### 4.3. Measurements on the Dorsoplantar Projection

Few hind limbs in equids have generally been analyzed morphometrically, and even fewer conformational studies of the hind hooves have been carried out on X-ray images in the dorsoplantar projection. The literature comparison will therefore mainly deal with the analogously determined measured values on the front hooves of donkeys.

Regarding the rather steeper and cubic appearance of the donkey hoof [2,3] compared to the horse hoof, the lateral and medial hoof wall angles (L/MWA) are expected to approach a right angle, as is a slight divergence of the coronet width (CW) compared to the foot width (FW). Respectively, the values measured in this population completely fulfill the expectations with average LWA and MWA between 87° and 88°, CW of 73 mm to 80 mm, and FW of 77 mm to 83 mm in length. El-Marakby et al. found almost identical lateral and medial hoof wall angles in the donkey population they studied, while significantly more acute angles were measured in El-Shafaey et al. [20,21]. Due to the conspicuous frequency of outlier values in this publication compared to other sources with consistent results, it can be assumed that the lateral hoof wall angles on the healthy donkey toe should be between 80 and 90°. The causes of which can only be speculated by influences such as previous shoeing and lack of previous hoof trimming, for example. When comparing the FW to examined horse hind hooves, there is a difference of about 20 to 30 mm, which suggests the more trapezoidal appearance of horse hooves compared to donkeys but cannot be proven without a corresponding measured width of the coronet band.

The lateral hoof wall length (LWL) was measured here at an average of 58 mm to 62 mm, the MWL at 57 mm to 61 mm; no relevant differences were found between medial and lateral or between left and right. The donkey populations studied by El-Marakby et al. and Wacker et al. showed comparable values [21,24], while Nocera et al. [19] measured slightly longer wall distances and El-Shafaey et al. [20] shorter wall distances. Except for Nocera et al. [19], the donkey hooves analyzed in the literature show an MWL that is on average a few millimeters longer. This observation was also made in ponies and warmbloods, although the differences between lateral and medial hoof wall length and coronet band height were only significant in the right forelimb of the pony population studied [9,11]. Overall, significant differences between lateral and medial measurements on the DP radiograph were rarely tested explicitly; thus, no reliable statement can be made on this issue. Based on the small differences in the data, however, the influence can be considered negligible, and minor asymmetries can be considered normal, provided they do not show extreme divergences that are relevant in the overall context in connection with a clinically occurring problem.

The measured lateral and medial coronet band height corresponds with the values provided for comparison at average dimensions of 57 to 61 mm and is almost identical to the variables LWL and MWL according to the angular orthogonal approximation of the lateral hoof walls [21].

As can be expected from the silhouette of the coffin bone, assuming a lateromedially balanced orientation in the hoof capsule, the lateral and medial sole thickness (LST, MST) show almost identical measured values of approximately 20 mm on average, while the sagittally measured sole is slightly thinner at 16 mm on average. Based on comparable results in another donkey population, while significantly shorter MST compared to LST were found in a pony population studied, it can be assumed that hoof-healthy donkeys tend to have a horizontally balanced orientation of the coffin bone and a strongly developed sole [11,21].

In the examined population of generally sound, lameness-free domesticated European donkeys, 10 cases of a coffin bone rotation (CR) more than 5° and 27 cases with a founder distance (FD) measured more than 20 mm on the hind limbs (right and left pooled) were detected. Minor deviations from the norm, which have not been sufficiently defined for the hind hooves of donkeys, do not automatically indicate a pathology like laminitis, for example. Such a condition should nevertheless be suspected and serve as a reason for the examiner to be able to better narrow down the probability of the presence of a disease with the help of a multi-parametric approach [18,35] in combination with the clinical examination. An important part of the clinical examination is the adspection, which is problematic in donkeys, as the detection of painful conditions is often unsuccessful due to their stoic nature or only successful in advanced pathological conditions [13]. In Great Britain, the prevalence of laminitis is reported to be around 13% of donkeys euthanized in one year [2]. Consequently, although no lameness in walk could be detected and general soundness, defined as showing physiological posture, behavior, nutritional and training condition, plus absent signs of an infectious disease and abnormal deviations in vital parameters [48,49], was confirmed, a pathological condition within the hoof cannot be excluded. It must therefore be considered that the inclusion of unrecognized donkeys suffering from chronic laminitis [3] in the study cannot be ruled out when evaluating the available measurement data. Collins et al. and Wacker et al. also came to similar conclusions [17,18,24]. In future studies and in everyday life, pain scores specially developed and successfully tested for donkeys can significantly simplify the detection of painful conditions for owners as well as for the treating veterinarians and farriers. It should be noted that although the EQUUS-DONKEY-COMPASS is better suited for the detection of orthopedic problems, painful conditions in the head area are better detected by the EQUUS_DONKEY-FAP. However, both systems were evaluated for acute pain of different localization and genesis, so the benefit of using them for chronic conditions is unclear [50].

### 4.4. Limitations

This study was mainly limited, despite the solid number of subjects, by a statistically relatively small sample size and the great presence of variation in the phenotype of the donkeys studied. Furthermore, measurement inaccuracies cannot be ruled out, as the interobserver agreement was not statistically analyzed. However, since the measurements were performed exclusively by two trained examiners, this influence is nevertheless considered to be low. In addition, mean values formed from repeated measurements would have increased the validity of the data. Kummer et al. used a software program in their study to evaluate a standardized X-ray procedure on the horse’s hoof and thus achieved excellent repeatability and low variance in the values determined [26]. Due to the frequent similarity of the measured values in the literature comparison and the frequently low standard deviation of the average measured values, it can nevertheless be assumed that the measurements tend to exhibit minor inaccuracies. Although a standardized procedure analogous to Kummer et al. [26] was used, minor projection-related inaccuracies in the measurements are also to be expected, but these cannot be avoided while dealing with living, non-sedated animals—especially donkeys. The measurements are also subject to the fundamental influence of the temporal relationship to the last hoof trimming and the influence of the farrier’s individual working method [8,9]. The trimming-associated error sources were reduced to a minimum in this case thanks to the clinic’s team of farriers, who are all trained equally and work according to the same method. Adding the senile age category to the juvenile and adult groups could provide better information on the possible correlation between the measured parameters and age. To calculate the influence of size, individual measured height at the withers would be more precise instead of the rough classification into size classes. In addition, a graduation based on the body condition score as a supplement to the weight allows a differentiation between a weight corresponding to the respective breed standard and an overweight animal. This differentiation can be used to gain further insights into the respective influence on the morphometric parameters, although all of the donkeys included in this study had a body condition score between two and four [3].

## 5. Conclusions

This study is the first to provide basic data on the radiographic normal conformation of the distal hindlimb in generally sound, lameness-free donkeys in two projections. Based on the measurement values obtained, the steeper hoof with its cubic appearance in both projections, the more distally located coffin bone in the hoof capsule that runs parallel to the dorsal hoof wall, and the strongly developed sole of the donkey can be confirmed in comparison to available measurement data on horses from other studies. Moreover, a broken-forward hoof pastern axis is often observed, but this can only be considered physiological if it is mild. A positive plantar angle is assumed to be desirable. Compared to existing data from forelimbs of donkeys, only slight differences can be assumed, but these can only be confirmed or refuted by further investigations. The absolute measured values show strong, significant correlations with the normalized values, so that the use of the absolute measured values is justifiable for reasons of practicability. Except for heel length and plantar coronet height, there are no relevant differences between the right and left hind limbs. No relevant correlations between the investigated variables and age can be demonstrated. In almost all cases, a positive correlation between the parameters and weight can be determined, with stronger correlations in the length measurements. When evaluating morphometric measurements, all proven influencing factors must be considered, i.e., the individually influenced factors such as height, weight/obesity, breed, and pre-existing disorders, as well as the externally influenced factors such as husbandry, including substrate, usage/workload and hoof care (farrier’s working method, trimming intervals). In the future, further studies of this type with larger sample numbers and evenly distributed groups regarding age, breed, height at the withers, and weight or BCS are required to determine and validate the factors influencing the measured values and the measurements per se more accurately. With the information obtained, measurements can be applied analogously to the procedure described here and then be evaluated in comparison to the available reference values. This helps to identify pathological conditions like laminitis, deviations in the hoof pastern axis, conformational abnormalities such as long toes and underrun heels or club feet, negative plantar angles, and imbalances, for example. These are commonly associated with damages caused by disproportions in pressure and tension, especially affecting the deep digital flexor tendon and the navicular bone.

## Figures and Tables

**Table 1:** Abbreviations for length measurements on the lateromedial radiograph.

		Abbr.	Parameter	Color	Definition
lm (90°)	l	DWL	Dorsal wall length	Pink	Distance of the hoof wall at the most dorsal point between the coronet band and the ground surface
		ST	Sole thickness	White	Vertical distance between the tip of the coffin bone and the sole
		DCH	Dorsal coronet height	Yellow	Vertical distance between the most dorsal point of the coronet to the ground surface
		HL	Heel length	Green	Distance of the heel between the most plantar point of the coronet and the ground surface
		PCH	Plantar coronet height	Orange	Vertical distance between the most plantar point of the coronet to the ground surface
		FD	Founder distance	Red	Vertical distance between the horizontal extension at the level of the dorsal coronet to the extensor process of the coffin bone
		SL	Sole length	Light blue	Distance between the intercept point of the dorsal hoof wall and of the heel at the ground surface

lm = lateromedial, l = length in mm, abbr. = abbreviation.

**Table 2:** Abbreviations for angular measurements on the lateromedial radiograph.

		Abbr.	Parameter	Color	Definition
lm (90°)	a	HWA	Hoof wall angle	Pink	Angle of the dorsal hoof wall to the ground surface
		CBA	Coffin bone angle	Red	Angle of the dorsal facies of the coffin bone to the ground surface
		CR_c	Coffin bone rotation calc.		Calculated angular deviation HWA and CBA
		CR_m	Coffin bone rotation meas.		Angle between the dorsal hoof wall and the dorsal facies of the coffin bone
		PA	Plantar angle	Light blue	Angle between the facies plantaris of the coffin bone and the ground surface
		HA	Heel angle	Green	Angle between HL and ground surface
		HPA1	Hoof pastern axis 1	Orange	Axial deviation in the proximal interphalangeal joint
		HPA2	Hoof pastern axis 2	Yellow	Axial deviation in the coffin joint

lm = lateromedial, a = angle in °, abbr. = abbreviation, calc. = calculated, meas. = measured.

**Table 3:** Abbreviations for length measurements on the dorsoplantar radiograph.

		Abbr.	Parameter	Color	Definition
dp (0°)	l	CW	Coronet width	Pink	Distance between lateral and medial coronet at widest point
		FW	Foot width	Red	Distance between distal end points of LWL and MWL
		LWL	Lateral wall length	Yellow	Distance between lateral coronet and ground surface in line with the lateral hoof wall
		MWL	Medial wall length	Orange	Distance between the medial coronet and the ground surface in line with the medial hoof wall
		LCH	Lateral coronet height	Light green	Vertical distance from the lateral coronet to the ground surface
		MCH	Medial coronet height	Dark green	Vertical distance from the medial coronet to the ground surface
		SST	Sagittal sole thickness	White	Vertical distance from sagittal coffin bone to the sole
		LST	Lateral sole thickness	Light blue	Vertical distance from lateral coffin bone to the sole
		MST	Medial sole thickness	Dark blue	Vertical distance from medial coffin bone to the sole

dp = dorsoplantar, l = length in mm, abbr. = abbreviation.

**Table 4:** Abbreviations for angular measurements on the dorsoplantar radiograph.

		Abbr.	Parameter	Color	Definition
dp (0°)	a	LWA	Lateral wall angle	Yellow	Angle between lateral hoof wall and ground surface
		MWA	Medial wall angle	Orange	Angle between medial hoof wall and ground surface

dp = dorsoplantar, a = angle in °, abbr. = abbreviation.

**Table 5:** Differences between right and left hind limb.

		v	*n*		Normally Distributed		*p* < 0.05 R vs. L	
			All	GAMD	All	GAMD	All	GAMD
lm (90°)	l	DWL	39	20	✓	✓	x	x
		DCH	38	19	✓	✓	x	x
		HL	34	19	✓	✓	*p* = 0.005	*p* = 0.002
		PCH	34	19	✓	✓	*p* = 0.008	*p* = 0.009
		FD	39	20	(✓)	✓	x	x
	a	HWA	39	20	✓	✓	x	x
		CBA	39	20	✓	✓	x	x
		CR_c	39	20	✓	x	x	x
		CR_m	39	20	(✓)	(✓)	x	x
		SA	39	20	✓	✓	x	x
		PA	39	20	✓	✓	x	x
		HA	39	20	✓	✓	x	x
		HPA2	39	20	(✓)	✓	x	x
dp (0°)	a	MWA	35	17	✓	✓	x	x
	l	CW	35	17	✓	✓	*p* = 0.04	*p* = 0.03
		FW	35	17	✓	✓	x	x
		LWL	35	17	✓	✓	x	x
		MWL	35	17	(✓)	(✓)	x	x
		LCH	35	17	✓	✓	x	x
		SST	34	16	/	✓	x	x
		LST	34	16	/	✓	x	x

lm = lateromedial, dp = dorsoplantar, l = length in mm, a = angle in °, v = variable, *n* = sample size, all = total population, ✓ = normally distributed in Shapiro–Wilk, Kolmogorov–Smirnov, Cramer–von Mises, and Anderson–Darling test, (✓) = normally distributed in majority of Shapiro–Wilk, Kolmogorov–Smirnov, Cramer–von Mises, and Anderson–Darling tests, / = not normally distributed, x = *p* > 0.05, GAMD = group of adult medium-sized donkeys.

**Table 6:** Correlation summary table weight and height.

	Correlation Coefficient rs	All	GAMD
Weight	+++ (0.7–1.0)	/	/
	++ (0.5–0.7)	DCH, SL, CW, FW, LWL, MWL, LCH, MCH	DCH, SL, FW
	+ (0.3–0.5)	DWL, FD, HWA, CBA, MST	DWL, FD, CBA, CW
	(+) (0.1–0.3)	ST, HL, PA, LST, SST	/
	(−) (−0.1–−0.3)	HPA 2	/
	−(−0.3–−0.5)	/	HPA2
Height	+++ (0.7–1.0)	/	not analyzed
	++ (0.5–0.7)	/	
	+ (0.3–0.5)	DWL, DCH, FD, SL HWA, CBA, CW, FW, LWL, MWL, LCH, MCH	
	(+) (0.1–0.3)	PA, MST	
	(−) (−0.1–−0.3)	HPA1	

rs = Spearman rank correlation coefficient, GAMD = group of adult medium-sized donkeys, abbreviations are listed in Table 1, Table 2, Table 3 and Table 4, +++ = very strong correlation, ++ = strong correlation, + = moderate strong correlation, (+) = weak correlation, (−) = weak negative correlation, − = moderate negative correlation.

## Data Availability

The original data presented in this study are included in the article in a summarized manner. Further inquiries on raw material can be directed to the corresponding author.

## Appendix A

**Table A1:** Normalized vs. absolute measurements in the total population.

Variable	*n*	Correlation with N (rs)	*p*	Systematic Difference	Proportional Difference	Linear Correlation	*p* (CUSUM)
DWL	53	+++ (0.96)	<0.0001	x	x	✓	0.92
ST	52	+++ (0.98)	<0.0001	x	x	✓	0.92
DCH	52	+++ (0.97)	<0.0001	x	x	✓	0.49
HL	49	+++ (0.99)	<0.0001	x	x	✓	0.68
PCH	49	+++ (0.99)	<0.0001	x	x	✓	0.89
FD	53	+++ (0.99)	<0.0001	x	x	✓	0.92

*n* = sample size, N = normalized data, rs = Spearman rank correlation coefficient, +++ = very strong correlation, x = no, ✓ = yes, CUSUM = CUSUM test.

**Table A2:** Normalized vs. absolute measurements in GAMD.

Variable	*n*	Correlation with N (rs)	*p*	Systematic Difference	Proportional Difference	Linear Correlation	*p* (CUSUM)
DWL	32	+++ (0.93)	<0.0001	x	x	✓	0.94
ST	31	+++ (0.98)	<0.0001	x	x	✓	0.93
DCH	31	+++ (0.96)	<0.0001	x	x	✓	0.93
HL	31	+++ (0.99)	<0.0001	x	x	✓	0.18
PCH	31	+++ (0.99)	<0.0001	x	x	✓	0.95
FD	32	+++ (0.96)	<0.0001	x	x	✓	0.99

*n* = sample size, N = normalized data, rs = Spearman rank correlation coefficient, +++ = very strong correlation, x = no, ✓ = yes, GAMD = group of adult medium-sized donkeys, CUSUM= CUSUM test.

**Table A3:** Absolute measurements right/left hindlimb, lateromedial 90°, total population.

		v	Side	*n*	*n Miss*	Mean	Std Dev	Min	Max	Median	LCL 95% for Mean	UCL 95% for Mean
lm 90°	l	DWL	R	40	6	83.93	21.51	59.41	169.21	77.27	77.05	90.81
			L	40	6	83.56	20.63	60.82	158.96	77.80	76.96	90.16
		ST	R	39	7	15.57	4.30	7.68	28.22	15.13	14.17	16.96
			L	38	7	15.16	4.09	7.70	24.71	14.44	13.84	16.49
		DCH	R	39	7	70.80	18.65	47.30	144.42	65.80	64.76	76.85
			L	39	7	70.04	18.45	44.06	140.96	64.62	64.05	76.02
		HL	R	36	10	17.96	5.72	8.46	30.31	17.22	16.03	19.90
			L	38	8	16.05	4.07	9.26	24.59	16.43	14.72	17.39
		PCH	R	36	10	14.38	4.87	5.57	24.40	12.91	12.73	16.02
			L	38	8	12.96	3.61	7.69	19.57	13.08	11.78	14.15
		FD	R	40	6	18.77	7.31	3.77	44.64	17.53	16.43	21.10
			L	40	6	18.02	7.73	2.87	46.13	17.17	15.55	20.49
		SL	R	40	6	114.36	32.20	80.98	254.43	103.75	104.06	124.66
			L	40	6	117.47	30.85	80.66	235.41	106.18	107.60	127.33
	a	HWA	R	40	6	55,72	4.31	46.95	69.65	55.34	54.34	57.10
			L	40	6	55.39	4.19	45.47	64.76	55.43	54.04	56.73
		CBA	R	40	6	56.67	4.70	47.24	66.78	57.16	55.16	58.17
			L	40	6	55.80	4.43	45.71	65.68	54.88	54.39	57.22
		CR-c	R	40	6	0.95	3.29	−7.15	7.84	0.43	−0.10	2.00
			L	40	6	0.42	3.01	−5.09	6.45	0.55	−0.54	1.38
		CR-m	R	40	6	1.26	3.27	−6.13	10.52	1.16	0.22	2.31
			L	40	6	1.32	2.92	−4.26	7.89	1.17	0.39	2.25
		PA	R	40	6	6.21	4.02	−5.15	13.76	6.17	4.92	7.49
			L	40	6	5.24	4.09	−4.38	14.65	4.96	3.93	6.55
		HA	R	40	6	52.81	5.81	35.10	61.68	52.73	50.32	54.04
			L	40	6	53.01	6.04	40.03	68.76	53.28	51.08	54.94
		HPA1	R	40	6	5.4	8.82	−17.98	23.99	6.00	2.58	8.22
			L	40	6	4.34	9.47	−26.50	18.33	6.98	1.31	7.37
		HPA2	R	40	6	−0,67	11.08	−38.50	18.75	−0,58	−4.21	2.88
			L	40	6	−3.14	10.11	−23.92	15.80	−2.54	−6.37	0.10

lm = lateromedial l = length in mm, a = angle in °, v = variable, *n* = sample size, *n miss* = missing samples, Std Dev = standard deviation, LCL = lower confidence interval limit, UCL = upper confidence interval limit.

**Table A4:** Absolute measurements right/left hindlimb, lateromedial 90°, GAMD.

		v	Side	*n*	*n Miss*	Mean	Std Dev	Min	Max	Median	LCL 95% for Mean	UCL 95% for Mean
lm (90°)	l	DWL	R	21	3	79.25	10.00	61.51	107.65	77.40	74.70	83.80
			L	21	3	77.82	7.67	61.94	94.63	75.86	74.33	81.31
		ST	R	20	4	15.78	3.60	10.08	23.95	14.60	14.09	17.46
			L	20	4	14.12	3.23	7.70	20.87	13.88	12.61	15.63
		DCH	R	20	4	67.90	9.59	51.34	92.86	66.57	63.41	72.39
			L	20	4	65.99	8.24	53.25	82.67	64.68	62.14	69.85
		HL	R	20	4	18.39	6.04	9.79	29.73	19.07	15.56	21.22
			L	21	3	15.58	4.06	10.10	23.96	14.58	13.73	17.42
		PCH	R	20	4	14.41	5.19	7.70	24.40	15.31	11.99	16.84
			L	21	3	12.32	3.81	7.69	19.57	11.48	10.58	14.05
		FD	R	21	3	17.46	3.73	10.16	27.30	16.75	15.76	19.16
			L	21	3	17.19	3.65	10.41	24.11	16.70	15.53	18.85
		SL	R	21	3	106.36	14.33	88.62	140.32	101.92	99.83	112.88
			L	21	3	108.38	13.43	86.43	133.48	105.58	102.27	114.49
	a	HWA	R	21	3	56.77	4.82	46.95	69.65	56.81	54.58	58.97
			L	21	3	56.46	3.68	51.12	64.51	56.41	54.79	58.13
		CBA	R	21	3	58.15	4.62	50.32	66.78	58.05	56.05	60.26
			L	21	3	56.95	4.13	52.13	65.68	55.83	55.07	58.83
		CR_c	R	21	3	1.38	3.58	−4.83	7.84	0.77	−0.25	3.01
			L	21	3	0.49	2.90	−3.74	6.45	0.46	−0.83	1.81
		CR_m	R	21	3	1.70	3.39	−6.13	10.52	0.94	0.16	3.24
			L	21	3	0.87	3.01	−4.26	7.89	1.16	−0.50	2.24
		PA	R	21	3	6.80	3.00	1.16	11.62	7.03	5.44	8.17
			L	21	3	5.64	3.80	0.93	14.65	5.51	3.91	7.37
		HA	R	21	3	52.17	5.89	35.10	61.68	52.17	49.49	54.86
			L	21	3	52.27	6.95	40.03	68.76	52.61	49.10	55.43
		HPA1	R	21	3	3.62	8.84	−17.98	16.89	4.20	−0.40	7.65
			L	21	3	2.04	10.48	−26.50	14.93	5.60	−2.73	6.81
		HPA2	R	21	3	−4.34	12.69	−38.50	17.67	−2.71	−10.12	1.43
			L	21	3	−6.14	8.70	−22.56	8.09	−5.34	−10.10	−2.18

lm= lateromedial, l = length in mm, a = angle in °, v = variable, *n* = sample size, *n miss* = missing samples, Std Dev = standard deviation, LCL = lower confidence interval limit, UCL = upper confidence interval limit, GAMD = group of adult medium-sized donkeys.

**Table A5:** Absolute measurements right/left hindlimb, dorsoplantar (0°), total population.

		v	Side	*n*	*n Miss*	Mean	Std Dev	Min	Max	Median	LCL 95% for Mean	UCL 95% for Mean
dp 0°	a	LWA	R	37	9	88.23	4.55	77.70	100.66	87.57	86.71	89.75
			L	35	11	87.22	4.41	80.02	97.74	86.45	85.71	88.74
		MWA	R	37	9	88.84	4.54	79.90	99.59	88.21	87.32	90.35
			L	35	11	88.64	4.10	80.43	96.96	88.48	87.23	90.04
	l	CW	R	37	9	80.33	24.95	53.63	182.56	72.05	72.01	88.64
			L	35	11	81.10	26.03	55.07	192.18	74.31	72.16	90.04
		FW	R	37	9	83.37	23.04	56.25	160.31	77.48	75.69	91.05
			L	35	11	83.65	23.67	56.29	176.04	76.94	75.52	91.78
		LWL	R	37	9	61.39	17.67	34.22	132.59	57.81	55.50	67.29
			L	35	11	62.26	16.53	39.17	130.21	59.08	56.58	67.94
		MWL	R	37	9	59.52	16.87	37.17	118.69	56.56	53.89	65.14
			L	35	11	61.02	19.19	34.26	139.66	57.81	54.43	67.61
		LCH	R	37	9	60.93	17.55	32.02	130.80	57.81	55.08	66.78
			L	35	11	61.94	17.12	38.27	129.42	58.86	56.05	67.82
		MCH	R	37	9	59.56	17.18	36.57	117.29	56.46	53.83	65.29
			L	35	11	59.85	18.64	33.77	138.95	57.30	53.45	66.26
		SST	R	37	9	16.71	6.00	7.95	34.61	15.51	14.71	18.71
			L	34	12	16.53	4.61	9.01	27.93	15.25	14.93	18.14
		LST	R	37	9	20.36	7.22	11.94	42.96	19.36	17.96	22.77
			L	34	12	19.53	5.88	10.54	38.83	18.65	17.48	21.58
		MST	R	37	9	19.80	6.12	9.55	35.61	18.83	17.76	21.84
			L	34	12	20.02	9.83	9.84	65.85	18.46	16.59	23.45

dp = dorsoplantar, l = length in mm, a = angle in °, v = variable, *n* = sample size, *n miss* = missing samples, Std Dev = standard deviation, LCL = lower confidence interval limit, UCL = upper confidence interval limit.

**Table A6:** Absolute measurements right/left hindlimb, dorsoplantar (0°), GAMD.

		v	Side	*n*	*n Miss*	Mean	StdDev	Min	Max	Median	LCL 95% for Mean	UCL 95% for Mean
dp(0°)	a	LWA	R	19	5	87.24	4.50	77.70	100.66	87.21	85.07	89.41
			L	17	7	86.63	4.35	81.28	97.74	86.33	84.39	88.87
		MWA	R	19	5	87.72	3.76	82.42	95.44	87.23	85.90	89.53
			L	17	7	88.04	3.58	83.37	96.31	87.37	86.20	89.88
	l	CW	R	19	5	73.93	11.39	53.63	104.36	72.05	68.44	79.42
			L	17	7	73.77	8.68	55.07	90.19	74.31	69.31	78.23
		FW	R	19	5	78.23	11.35	57.22	105.58	77.48	72.76	83.70
			L	17	7	78.07	9.40	59.65	95.92	76.94	73.24	82.91
		LWL	R	19	5	58.38	5.63	45.07	68.55	57.81	55.67	61.10
			L	17	7	58.67	6.58	39.17	67.00	59.21	55.28	62.05
		MWL	R	19	5	57.37	6.52	42.99	67.93	57.30	54.23	60.51
			L	17	7	56.21	7.65	34.26	66.34	57.11	52.27	60.14
		LCH	R	19	5	58.02	5.42	44.70	67.79	57.81	55.41	60.64
			L	17	7	57.84	6.63	38.27	65.72	59.16	54.43	61.25
		MCH	R	19	5	57.92	9.05	42.67	85.85	56.76	53.56	62.29
			L	17	7	54.91	7.26	33.77	65.85	55.93	51.17	58.64
		SST	R	19	5	16.29	4.20	10.97	25.49	15.23	14.27	18.31
			L	16	8	16.10	3.13	11.39	22.71	15.25	14.43	17.77
		LST	R	19	5	19.73	4.53	13.44	30.08	19.61	17.55	21.92
			L	16	8	18.88	3.57	12.73	26.89	18.65	16.97	20.78
		MST	R	19	5	19.58	4.02	15.13	29.09	18.65	17.64	21.51
			L	18	8	21.40	12.19	13.27	65.85	19.04	14.90	27.90

dp = dorsoplantar, l = length in mm, a = angle in °, v = variable, *n* = sample size, *n miss* = missing samples, Std Dev = standard deviation, LCL = lower confidence interval limit, UCL = upper confidence interval limit, GAMD = group of adult medium-sized donkeys.

## References

[1] Dai F., Segati G., Brscic M., Chincarini M., Costa E.D., Ferrari L., Burden F., Judge A., Minero M. (2018). Effects of Management Practices on the Welfare of Dairy Donkeys and Risk Factors Associated with Signs of Hoof Neglect. J. Dairy Res..

[2] Thiemann A., Rickards K. (2013). Donkey Hoof Disorders and Their Treatment. Practice.

[3] Evans L., Crane M., Preston E. (2021). The Clinical Companion of the Donkey.

[4] Morrow L.D., Smith K.C., Piercy R.J., du Toit N., Burden F.A., Olmos G., Gregory N.G., Verheyen K.L.P. (2011). Retrospective Analysis of Post-Mortem Findings in 1,444 Aged Donkeys. J. Comp. Pathol..

[5] Reix Nèe Broster C.E., Burn C.C., Pritchard J.C., Barr A.R.S., Whay H.R. (2014). The Range and Prevalence of Clinical Signs and Conformation Associated with Lameness in Working Draught Donkeys in Pakistan. Equine Vet. J..

[6] Hassanpour A., Dehghani S.N. (2012). Hoof Morphometry before and after Trimming in Donkeys. Res. Opin. Anim. Vet. Sci..

[7] O’Grady S.E. (2008). Basic Farriery for the Performance Horse. Vet. Clin. N. Am. Equine Pract..

[8] Kummer M., Gygax D., Lischer C., Auer J. (2009). Comparison of the Trimming Procedure of Six Different Farriers by Quantitative Evaluation of Hoof Radiographs. Vet. J..

[9] Kummer M., Geyer H., Imboden I., Auer J., Lischer C. (2006). The Effect of Hoof Trimming on Radiographic Measurements of the Front Feet of Normal Warmblood Horses. Vet. J..

[10] Cripps P.J., Eustace R.A. (1999). Radiological Measurements from the Feet of Normal Horses with Relevance to Laminitis. Equine Vet. J..

[11] Thieme K., Ehrle A., Lischer C. (2015). Radiographic Measurements of the Hooves of Normal Ponies. Vet. J..

[12] Vilsmeier A. (2004). Untersuchungen zur Hufform und zum Hornwachstum beim Esel (*Equus asinus*). Doctoral Thesis.

[13] Bartmann C.P., Pietta D., Litzke L.F. (2020). Hufpflege und Hufbeschlag bei Esel und Maultier. Der Huf. Lehrbuch des Hufbe- Schlags.

[14] Burden F.T.A. (2015). Donkeys Are Different. J. Equine Vet. Sci..

[15] Burnham S. Anatomical Differences of the Donkey and Mule. Proceedings of the 2002 American Association of Equine Practitioners Convention.

[16] Wissdorf H., Jerbi H., Fürst A. (2024). Unterschiede in der Anatomie von Esel/Muli und Pferd.

[17] Wacker J., Schaus K., Büttner K., Röcken M., Bartmann C.P. (2024). Incidence of Radiological Changes of the Coffin Bone in Donkeys in Consideration of the Age. Pferdeheilkunde.

[18] Collins S.N., Dyson S.J., Murray R.C., Burden F., Trawford A. (2011). Radiological Anatomy of the Donkey’s Foot: Objective Characterisation of the Normal and Laminitic Donkey Foot. Equine Vet. J..

[19] Nocera I., Aliboni B., Puccinelli C., Pietrini G., Sgorbini M., Citi S., Ricardi G. (2020). Radiographic Parameters of the Digit in a Cohort Population of Amiata Donkeys. Open Vet. J..

[20] El-Shafaey E.A., Salem M.G., Mosbah E., Zaghloul A.E. (2017). Morphometric Evaluation of Relevant Radiographic Parameters of the Forefeet of Clinically Normal Donkeys (*Equus asinus*). J. Hell. Vet. Med. Soc..

[21] El-Marakby A.I., Abdelgalil A.I., Metwally A.A., Mostafa M.B., Soliman A.S. (2024). Photometric and Radiometric Hoof Capsule Evaluation in Normal and Long Toeunderrun Heel in Donkeys. J. Hell. Vet. Med. Soc..

[22] El-Marakby A.I., Mostafa M.B., Metwally A.A., Soliman A.S., Abdelgalil A.I. (2024). Distal Phalanx Radiographic Grading Features in Long-Toe, Underrun Heel in Egypt Baladi Donkeys (*Equus asinus*). Equine Vet. Educ..

[23] El-Marakby A., Abdelgalil A., Mostafa M., Soliman A. (2023). Relationships between the Shape of the Hoof Capsule and Orientation of the Distal Phalanx in Long Toe Underrun Heels in Donkeys. Equine Vet. Educ..

[24] Wacker J., Schaus K., Jandowsky A., Büttner K., Röcken M., Bartmann C.P. (2024). Radiographic Measurements of the Hoof in Generally Sound Donkeys with Emphasis on the Front Limbs. Front. Vet. Sci..

[25] Kalka K., Pollard D., Dyson S.J. (2021). An Investigation of the Shape of the Hoof Capsule in Hindlimbs, Its Relationship with the Orientation of the Distal Phalanx and Comparison with Forelimb Hoof Capsule Conformation. Equine Vet. Educ..

[26] Kummer M., Lischer C., Ohlerth S., Vargas J., Auer J. (2004). Evaluation of a Standardised Radiographic Technique of the Equine Hoof. Schweiz. Arch. Tierheilkd..

[27] Kotoyori Y., Endo Y., Murase H., Sato F., Korosue K. (2024). Changes in Aspects of Hoof and Distal Limb Conformation in Foals by Radiographic Evaluation. J. Vet. Med. Sci..

[28] Khan R.Z.U., Rosanowski S.M., Parkes R.S.V. (2023). Hoof Morphometry in a Population of Lame and Nonlame Working Donkeys in Pakistan. Equine Vet. J..

[29] Leśniak K., Whittington L., Mapletoft S., Mitchell J., Hancox K., Draper S., Williams J. (2019). The Influence of Body Mass and Height on Equine Hoof Conformation and Symmetry. J. Equine Vet. Sci..

[30] Mostafa M.B., Abdelgalil A.I., Farhat S.F., Raw Z., Kubasiewicz L.M. (2020). Morphometric Measurements of the Feet of Working Donkeys Equus Asinus in Egypt. J. Equine Sci..

[31] Sefton A., Haseler C.J., Jarvis G.E., Scott V.H.L., Mcgovern K.F. (2019). Laminitis in Donkeys: A Pilot Study Investigating Radiographic versus Post-Mortem Measurements. Equine Vet. J..

[32] Misk N.A., Hifny A. (1983). Anatomy of the Hoof in Donkeys. Assiut Vet. Med. J..

[33] Thiemann A.K., Poore L.A. (2019). Hoof Disorders and Farriery in the Donkey. Vet. Clin. N. Am. Equine Prac..

[34] Thieme K., Ehrle A., Lischer C. (2015). Morphometrische Messungen Am Pferdehuf-Eine Literaturübersicht. Pferdeheilkunde.

[35] Collins S.N., Dyson S.J., Murray R.C., Newton J.R., Burden F., Trawford A.F. (2012). Development of a Quantitative Multivariable Radiographic Method to Evaluate Anatomic Changes Associated with Laminitis in the Forefeet of Donkeys. Am. J. Vet. Res..

[36] Walker M., Taylor T., Slater M., Hood D., Weir V., Elslander J. (1995). Radiographic Appearance of the Feet of Mammoth Donkeys and the Finding of Subclinical Laminitis. Vet. Radiol. Ultrasound.

[37] Herbrecht V., Waldern N.M., Mikkelsen S.E., Kjaer M., Dittmann M.T., Wiestner T., Weishaupt M.A. (2020). Hoof Conformation in Icelandic Competition Horses and Its Interrelationship with Hoof Pathologies and Tölt Performance. Vet. J..

[38] Cardona G.A., Uribe A., Ortved K. (2021). Determination of Positional Parameters of the Distal Phalanx Within the Hoof Capsule in Sound Colombian Paso Horses. J. Equine Vet. Sci..

[39] McGreevy P.D., Rogers L.J. (2005). Motor and Sensory Laterality in Thoroughbred Horses. Appl. Anim. Behav. Sci..

[40] Sharp Y., Tabor G. (2022). An Investigation into the Effects of Changing Dorso-Plantar Hoof Balance on Equine Hind Limb Posture. Animals.

[41] Mellish M., Lucas Z., Lancaster L., Stull J., Floyd A. (2023). Visual and Morphometric Description of Feral Horse Hooves from Sable Island National Park Reserve. Can. Vet. J..

[42] White J., Mellor D., Duz M., Lischer C., Voute L. (2008). Diagnostic Accuracy of Digital Photography for the Measurement of Foot Conformation in the Horse. Equine. Vet. J..

[43] Sellke L., Patan-Zugaj B., Ludewig E., Cimrman R., Witter K. (2023). Comparison of Six Different Methods for Measuring the Equine Hoof and Recording of Its Three-Dimensional Conformation. J. Equine Vet. Sci..

[44] Dyson S.J., Tranquille C.A., Collins S.N., Parkin T.D.H., Murray R.C. (2011). An Investigation of the Relationships between Angles and Shapes of the Hoof Capsule and the Distal Phalanx. Equine Vet. J..

[45] Thiemann A.K., Buil J., Rickards K., Sullivan R.J. (2022). A Review of Laminitis in the Donkey. Equine Vet. Educ..

[46] Hampson B.A., de Laat M.A., Mills P.C., Walsh D.M., Pollitt C.C. (2013). The Feral Horse Foot. Part B: Radiographic, Gross Visual and Histopathological Parameters of Foot Health in 100 Australian Feral Horses. Aust. Vet. J..

[47] Pezzanite L., Bass L., Kawcak C., Goodrich L., Moorman V. (2019). The Relationship between Sagittal Hoof Conformation and Hindlimb Lameness in the Horse. Equine Vet. J..

[48] Glitz F., Deegen E., Wissdorf H., Gerhards H., Huskamp B., Deegen E. (2010). Allgemeine Untersuchung. Praxisorientierte Anatomie und Propädeutik des Pferdes.

[49] Bartmann C.P., Gehlen H., Brehm W., Gehlen H., Ohnesorge B., Wehrend A. (2016). Klinische Untersuchung. Handbuch Pferdepraxis.

[50] van Dierendonck M.C., van Loon J.P.A.M., Burden F.A., Rickards K. (2020). Monitoring Acute Pain in Donkeys with the Equine Utrecht University Scale for Donkeys Composite Pain Assessment (Equus-Donkey-Compass) and the Equine Utrecht University Scale for Donkey Facial Assessment of Pain (Equus-Donkey-Fap). Animals.

